# Supplementary material for: House design and risk of malaria, acute respiratory infection and gastrointestinal illness in Uganda: A cohort study
Source: PLOS Glob Public Health. 2022 Mar 3;2(3):e0000063. doi: 10.1371/journal.pgph.0000063 (PMC10022195; doi:10.1371/journal.pgph.0000063)
Supplement: S1 Table — (DOCX) [file pgph.0000063.s001.docx]

**Table S1.** Diagnoses included in the definitions of acute respiratory infection and gastrointestinal illness

| **Acute respiratory, eye, ear, nose and throat infection** | **Gastrointestinal and related infection** |
| --- | --- |
| Asthma Exacerbation | Abdominal pain - unspecified |
| Common cold / flu | Diarrhoea - acute |
| Conjunctivitis | Dysentery |
| Otitis externa | Dyspepsia / gastritis |
| Otitis media - nonsuppurative | Gastroenteritis |
| Otitis media - suppurative | Helminth infection |
| Pharyngitis | Peptic ulcer disease |
| Pneumonia | Visceral larva migrans |
| Pneumonia – severe |  |
| Respiratory failure |  |
| Stye |  |
| Tonsillitis |  |
| Tuberculosis - pulmonary |  |
| Upper respiratory tract infection (URTI) |  |
