# Supplementary material for: House design and risk of malaria, acute respiratory infection and gastrointestinal illness in Uganda: A cohort study
Source: PLOS Glob Public Health. 2022 Mar 3;2(3):e0000063. doi: 10.1371/journal.pgph.0000063 (PMC10022195; doi:10.1371/journal.pgph.0000063)
Supplement: S2 Table — (DOCX) [file pgph.0000063.s002.docx]

**Table S2.** Association between room characteristics and malaria vector density in Nagongera, Uganda (unadjusted results)

| Characteristic | | HBR ^a^ (total collection nights) | IRR (95% CI) | p value |
| --- | --- | --- | --- | --- |
| ***Household-level characteristics*** | | | | |
| Wealth category | Poorest | 1.91 (2342) | 1 | 0.71 |
|  | Middle | 2.09 (2445) | 1.30 (0.65, 2.60) |  |
|  | Least poor | 2.18 (2844) | 1.15 (0.70, 1.90) |  |
| IRS in the past 12 months | No | 2.46 (635) | 1 | 0.77 |
|  | Yes | 2.03 (6996) | 0.90 (0.46, 1.77) |  |
| People per bedroom | ≥3 people | 2.11 (5013) | 1 | 0.76 |
|  | 0-2 people | 2.00 (2618) | 0.92 (0.54, 1.56) |  |
| ***Room-level characteristics*** | | | | |
| LLIN use in the room | <50% of nights | 2.38 (3855) | 1 | 0.12 |
|  | ≥50% of nights | 1.75 (3776) | 0.70 (0.44, 1.10) |  |
| House type ^b^ | Traditional | 2.98 (3251) | 1 | <0.001 |
|  | Modern | 1.39 (4380) | 0.44 (0.29, 0.68) |  |
| Main roof material | Thatched | 3.75 (1398) | 1 | 0.006 |
|  | Metal | 1.69 (6233) | 0.41 (0.22, 0.77) |  |
| Type of eaves | Open | 3.68 (1602) | 1 | 0.003 |
|  | Closed | 1.64 (6029) | 0.41 (0.23, 0.74) |  |
| Windows present | No | 2.41 (4083) | 1 | 0.08 |
|  | Yes | 1.67 (3548) | 0.67 (0.43, 1.04) |  |
| Airbricks present | No | 2.89 (3351) | 1 | 0.001 |
|  | Yes | 1.43 (4280) | 0.48 (0.31, 0.73) |  |
| Average time to bed | 2100 or later | 2.16 (2626) | 1 | 0.99 |
|  | Before 2100 | 2.02 (5005) | 1.00 (0.76, 1.32) |  |
| CI: confidence interval, HBR: Human biting rate, IRR: Incidence rate ratio, IRS: Indoor residual spraying, LLIN: Long-lasting insecticide-treated net | | | | |
| ^a^ Total adult female *Anopheles* caught per room per night | | |  |  |
| ^b^ Modern houses: closed eaves, brick (not mud walls), metal (not thatched) roof; traditional houses: all other houses | | | | |
