# Supplementary material for: House design and risk of malaria, acute respiratory infection and gastrointestinal illness in Uganda: A cohort study
Source: PLOS Glob Public Health. 2022 Mar 3;2(3):e0000063. doi: 10.1371/journal.pgph.0000063 (PMC10022195; doi:10.1371/journal.pgph.0000063)
Supplement: S3 Table — (DOCX) [file pgph.0000063.s003.docx]

**Table S3.** Association between house type and malaria in Nagongera, Uganda (unadjusted results)

| Characteristic | | PR (total blood slides) | OR ^a^ (95% CI) | p  value | Incidence  (total person years) | IRR ^a^ (95% CI) | p value |
| --- | --- | --- | --- | --- | --- | --- | --- |
| ***Individual-level characteristics*** | | | | |  |  |  |
| Gender | Female | 7.8 (6901) | 1 | 0.03 | 0.04 (498.9) | 1 | 0.45 |
|  | Male | 12.8 (6326) | 1.72 (1.05, 2.82) |  | 0.03 (459.2) | 0.79 (0.43, 1.45) |  |
| Age ^a^ | <5 years | 3.9 (3663) | 1 | <0.001 | 0.04 (249.0) | 1 | 0.07 |
|  | 5-15 years | 13.9 (5450) | 3.96 (2.30, 6.83) |  | 0.05 (402.7) | 1.18 (0.59, 2.37) |  |
|  | >15 years | 10.8 (4114) | 2.95 (1.60, 5.43) |  | 0.02 (306.4) | 0.44 (0.16, 1.21) |  |
| ITN use the previous night | No | 9.2 (563) | 1 | 0.66 | - | - | - |
|  | Yes | 9.9 (12133) | 1.08 (0.76, 1.54) |  | - | - |  |
| ITN use | <90% of clinic visits | - | - | - | 0.03 (104.8) | 1 | 0.50 |
|  | ≥90% of visits | - | - |  | 0.04 (853.3) | 1.43 (0.51, 4.06) |  |
| ***Household-level characteristics*** | | | | |  |  |  |
| Wealth category | Poorest | 11.1 (4506) | 1 | 0.81 | 0.05 (326.2) | 1 | 0.15 |
|  | Middle | 9.1 (4266) | 0.80 (0.41, 1.56) |  | 0.05 (308.7) | 0.87 (0.43, 1.77) |  |
|  | Least poor | 10.2 (4455) | 0.91 (0.45, 1.84) |  | 0.02 (323.2) | 0.42 (0.17, 1.04) |  |
| IRS in the past 12 months | No | 7.1 (1418) | 1 | 0.29 | 0.04 (101.4) | 1 | 0.99 |
|  | Yes | 10.6 (11809) | 1.56 (0.69, 3.52) |  | 0.04 (856.7) | 1.01 (0.36, 2.78) |  |
| House type ^b^ | Traditional | 9.8 (7169) | 1 | 0.74 | 0.04 (517.8) | 1 | 0.65 |
|  | Modern | 10.7 (6058) | 1.10 (0.62, 1.94) |  | 0.04 (440.3) | 0.86 (0.44, 1.67) |  |
| Main roof material | Thatched | 8.0 (3516) | 1 | 0.25 | 0.04 (254.6) | 1 | 0.75 |
|  | Metal | 11.0 (9711) | 1.42 (0.79, 2.56) |  | 0.04 (703.5) | 0.89 (0.42, 1.86) |  |
| Type of eaves | Open | 8.9 (3885) | 1 | 0.47 | 0.04 (281.0) | 1 | 0.77 |
|  | Closed | 10.7 (9342) | 1.23 (0.70, 2.15) |  | 0.04 (677.1) | 0.90 (0.44, 1.83) |  |
| Windows per room | 0 window | 7.3 (5574) | 1 | 0.09 | 0.04 (406.5) | 1 | 0.40 |
|  | 0<1 window | 12.8 (4159) | 1.85 (1.04, 3.29) |  | 0.03 (298.8) | 0.64 (0.27, 1.53) |  |
|  | 1 window | 11.6 (3494) | 1.66 (0.79, 3.47) |  | 0.05 (252.8) | 1.23 (0.58, 2.60) |  |
| Airbricks present | No | 7.9 (5300) | 1 | 0.18 | 0.03 (382.8) | 1 | 0.33 |
|  | Yes | 11.7 (7927) | 1.53 (0.82, 2.86) |  | 0.05 (575.3) | 1.44 (0.69, 3.00) |  |
| People per bedroom | ≥3 people | 11.1 (9560) | 1 | 0.24 | 0.04 (693.3) | 1 | 0.59 |
|  | 0-2 people | 7.9 (3667) | 0.69 (0.37, 1.28) |  | 0.03 (264.8) | 0.81 (0.38, 1.73) |  |

CI: confidence interval, IRS: indoor residual spraying, ITN: insecticide treated net, OR: odds ratio, PR: parasite rate

^a^ Malaria prevalence: age at the time of the routine clinic visit; malaria incidence: mean age during follow up

^b^ Modern houses: closed eaves, brick (not mud walls), metal (not thatched) roof; traditional houses: all other houses
