# Supplementary material for: House design and risk of malaria, acute respiratory infection and gastrointestinal illness in Uganda: A cohort study
Source: PLOS Glob Public Health. 2022 Mar 3;2(3):e0000063. doi: 10.1371/journal.pgph.0000063 (PMC10022195; doi:10.1371/journal.pgph.0000063)
Supplement: S4 Table — (DOCX) [file pgph.0000063.s004.docx]

**Table S4.** Association between house type and incidence of acute respiratory infection and diarrhoeal disease in Nagongera, Uganda (unadjusted results)

| Characteristic | | ARI | | | Gastrointestinal illness | | |
| --- | --- | --- | --- | --- | --- | --- | --- |
|  |  | Incidence (total person years) | IRR (95% CI) | p value | Incidence (total person years) | IRR (95% CI) | p value |
| Gender | Female | 2.8 (498.9) | 1 | 0.06 | 0.5 (498.9) | 1 | 0.02 |
|  | Male | 2.5 (459.2) | 0.90 (0.81, 1.00) |  | 0.3 (459.2) | 0.69 (0.51, 0.93) |  |
| Mean age during follow up | <5 years | 4.3 (249.0) | 1 | <0.001 | 0.5 (249.0) | 1 | <0.001 |
|  | 5-15 years | 2.2 (402.7) | 0.51 (0.46, 0.56) |  | 0.1 (402.7) | 0.23 (0.16, 0.33) |  |
|  | >15 years | 2.0 (306.4) | 0.47 (0.41, 0.54) |  | 0.7 (306.4) | 1.25 (0.93, 1.69) |  |
| Wealth category | Poorest | 2.6 (326.2) | 1 | 0.12 | 0.4 (326.2) | 1 | 0.89 |
|  | Middle | 3.0 (308.7) | 1.16 (0.99, 1.36) |  | 0.4 (308.7) | 1.10 (0.74, 1.63) |  |
|  | Least poor | 2.5 (323.2) | 0.97 (0.79, 1.19) |  | 0.4 (323.2) | 1.05 (0.72, 1.52) |  |
| House type ^a^ | Traditional | 2.8 (517.8) | 1 | 0.29 | 0.5 (517.8) | 1 | 0.03 |
|  | Modern | 2.5 (440.3) | 0.92 (0.79, 1.07) |  | 0.3 (440.3) | 0.72 (0.55, 0.96) |  |
| Windows per room | 0 window | 2.6 (406.5) | 1 | 0.71 | 0.4 (406.5) | 1 | 0.004 |
|  | 0<1 window | 2.8 (298.8) | 1.07 (0.90, 1.27) |  | 0.5 (298.8) | 1.16 (0.82, 1.64) |  |
|  | 1 window | 2.6 (252.8) | 1.01 (0.81, 1.26) |  | 0.3 (252.8) | 0.71 (0.51, 0.99) |  |
| Airbricks present | No | 2.6 (382.8) | 1 | 0.74 | 0.5 (382.8) | 1 | 0.17 |
|  | Yes | 2.7 (575.3) | 1.03 (0.88, 1.21) |  | 0.4 (575.3) | 0.80 (0.59, 1.10) |  |
| Main floor material | Earth, sand or dung | 2.7 (867.0) | 1 | 0.77 | 0.4 (867.0) | 1 | 0.96 |
|  | Cement or concrete | 2.6 (91.1) | 0.96 (0.73, 1.27) |  | 0.4 (91.1) | 1.01 (0.65, 1.57) |  |
| People per bedroom | ≥3 people | 2.6 (693.3) | 1 | 0.50 | 0.4 (693.3) | 1 | 0.65 |
|  | 0-2 people | 2.8 (264.8) | 1.06 (0.90, 1.25) |  | 0.4 (264.8) | 1.07 (0.79, 1.45) |  |
| Sanitation facility ^b^ | Unimproved | 2.6 (878.3) | 1 | 0.09 | 0.4 (878.3) | 1 | 0.01 |
|  | Improved | 3.5 (79.8) | 1.35 (0.96, 1.90) |  | 0.7 (79.8) | 1.77 (1.12, 2.79) |  |
| Drinking water source ^c^ | Unimproved | 2.8 (330.3) | 1 | 0.22 | 0.4 (330.3) | 1 | 0.26 |
|  | Improved | 2.6 (627.8) | 0.91 (0.79, 1.06) |  | 0.4 (627.8) | 0.84 (0.62, 1.14) |  |

ARI: acute respiratory infection, CI: confidence interval, ITN: long-lasting insecticide treated net, IRR: incidence rate ratio, IRS: indoor residual spraying

^a^ Modern houses: closed eaves, brick (not mud walls), metal (not thatched) roof; traditional houses: all other houses

^b^ Sanitation facility was defined as improved or unimproved using WHO Joint Monitoring Programme (WHO-JMP) criteria which consider whether or not a sanitation facility adequately separates human excreta from human contact (improved toilets include latrines with washable slabs)^25^

^c^ Drinking water source was defined as improved or unimproved using WHO Joint Monitoring Programme (WHO-JMP) criteria which consider whether or not a drinking water source has adequate protection from outside contamination (improved sources include piped water, protected wells and rainwater).^25^
