# Supplementary material for: House design and risk of malaria, acute respiratory infection and gastrointestinal illness in Uganda: A cohort study
Source: PLOS Glob Public Health. 2022 Mar 3;2(3):e0000063. doi: 10.1371/journal.pgph.0000063 (PMC10022195; doi:10.1371/journal.pgph.0000063)
Supplement: Attachment — Submitted filename: Rebuttal letter 13.10.21.docx [file pgph.0000063.s007.docx]

Dear Dr Atulomah,

**Re: PGPH-D-21-00703. House design and risk of malaria, acute respiratory infection and gastrointestinal illness in Uganda: a cohort study**

Thank you for your email dated 8^th^ October 2021 and for the efforts of the three referees who reviewed our work. We were delighted with the overall positive endorsement of our submission. We submit a revised version of the manuscript in which we have incorporated changes based upon the editor’s and reviewers’ requests. These changes and our responses are listed in black text and the editor/reviewer comments in blue text, with line numbers referring to the tracked changes manuscript.

**Journal Requirements:**

1. Please include a complete copy of PLOS’ questionnaire on inclusivity in global research in your revised manuscript. Our policy for research in this area aims to improve transparency in the reporting of research performed outside of researchers’ own country or community. The policy applies to researchers who have travelled to a different country to conduct research, research with Indigenous populations or their lands, and research on cultural artefacts. The questionnaire can also be requested at the journal’s discretion for any other submissions, even if these conditions are not met.  Please find more information on the policy and a link to download a blank copy of the questionnaire here: [https://journals.plos.org/globalpublichealth/s/best-practices-in-research-reporting](https://protect-eu.mimecast.com/s/mP4vC8EVLUwnNgGsnegmX?domain=journals.plos.org). Please upload a completed version of your questionnaire as Supporting Information when you resubmit your manuscript.

We thank the editors for their request and have included this questionnaire in our resubmission.

2. Please provide separate figure files in .tif or .eps format only, and remove any figures embedded in your manuscript file.  If you are using LaTeX, you do not need to remove embedded figures.

For more information about figure files please see our guidelines:

<https://journals.plos.org/globalpublichealth/s/figures>

We confirm that the manuscript contains no embedded figures and include figure files in the requested format.

3. As the link do not return a result, please amend your Data Availability Statement and indicate where the data may be found.

We thank the editors for their comment. We have corrected the link in the Data Availability Statement.

4. Please provide us with a direct link to the base layer of the map used in Figure 2 and ensure this location is also included in the figure legend.

Please note that, because all PLOS articles are published under a CC BY license ([creativecommons.org/licenses/by/4.0/](http://protect-eu.mimecast.com/s/vYaxC0LKzCr6Nzxh2r8Qu?domain=creativecommons.org)), we cannot publish proprietary maps such as Google Maps, Mapquest or other copyrighted maps. If your map was obtained from a copyrighted source please amend the figure so that the base map used is from an openly available source.

Please note that only the following CC BY licences are compatible with PLOS licence: CC BY 4.0, CC BY 2.0  and CC BY 3.0, meanwhile such licences as CC BY-ND 3.0 and others are not compatible due to additional restrictions. If you are unsure whether you can use a map or not, please do reach out and we will be able to help you.

The following websites are good examples of where you can source open access or public domain maps:

* U.S. Geological Survey (USGS) - All maps are in the public domain. ([http://www.usgs.gov](http://protect-eu.mimecast.com/s/rTEvCg204iYVEJ3T3nRtE?domain=usgs.gov))

* PlaniGlobe - All maps are published under a Creative Commons license so please cite “PlaniGlobe, [http://www.planiglobe.com](http://protect-eu.mimecast.com/s/4JDICj814Cypo0rs17kOD?domain=planiglobe.com), CC BY 2.0” in the image credit after the caption. ([http://www.planiglobe.com/?lang=enl](http://protect-eu.mimecast.com/s/-eGeCk714Tqm2vET8CIob?domain=planiglobe.com))

* Natural Earth - All maps are public domain. ([http://www.naturalearthdata.com/about/terms-of-use/](http://protect-eu.mimecast.com/s/SbFSCl714TzgRlruVN_-Z?domain=naturalearthdata.com))

We have amended Figure 2 with a base map from an openly available source (http://maps.stamen.com/). We have added a link to this open-source base map in the legend to Figure 2.

5. Please ensure that the funders and grant numbers match between the Financial Disclosure field and the Funding Information tab in your submission form. Note that the funders must be provided in the same order in both places as well.

We have attended to this.

Please review your reference list to ensure that it is complete and correct. If you have cited papers that have been retracted, please include the rationale for doing so in the manuscript text, or remove these references and replace them with relevant current references. Any changes to the reference list should be mentioned in the rebuttal letter that accompanies your revised manuscript. If you need to cite a retracted article, indicate the article’s retracted status in the References list and also include a citation and full reference for the retraction notice.

We confirm that our reference list is complete and correct. **Peer review comments:**

Reviewer #1: 1. From Lines 5 to 6, in the abstract section, "Data were analysed from a cohort study of child and adult residents (n=531)". The adult mentioned here, are they mother/child pairs or father/child pairs or both male and female adults?

We thank the reviewer for their comment and confirm that both male and female adults were included. This information is given in the Methods (Line 105) section which states “*All permanent residents of enrolled households, regardless of age, were screened and enrolled in the cohort study…”.* However, to aid clarity, we have updated Lines 5-6 in the Abstract to:

*“Data were analysed from a cohort study of male and female child and adult residents”*

2. In Line 10, on measured gastrointestinal illness, was water in the study areas assessed for heavy metal metals, microorganisms and permissible chemical standards?

We thank the reviewer for their comment; these exposures were not assessed in our study.

3. In line 11, what is the provision for drawing blood samples from participants in terms of ethical issues relating to evasive procedure?

We thank the reviewer for their comment and confirm that all correct ethical approvals, participant sensitisation and consent were done and were approved in advance by the Uganda National Council for Science and Technology; Makerere University School of Medicine Research and Ethics Committee; University of California, San Francisco Committee for Human Research; Department of Biosciences Ethics Committee, Durham University; and the London School of Hygiene & Tropical Medicine Ethics Committee (Lines 191-194).

To aid clarity over consent we have updated the manuscript to read (Lines 185-190):

*“Written informed consent was obtained in the appropriate language from guardians of children and from adult participants and assent was requested from children 8-17 years of age as per Uganda National Council for Science and Technology guidelines. Written informed consent and assent forms were available in all local languages prevalent in Tororo district (English, Japadhola, Kiswahili, Lusamya and Luganda). If the adult, child, or parent/guardian was unable to read, an independent witness was identified.”*

4. In Lines 19 and 20, belief systems, culture, and geographical locations are essential factors that must be considered. This recommendation should be location-specific.

We thank the reviewer and concur with their comment; hence we state in Lines 20-21 that there is a need to assess effectiveness of housing improvements “*across a range of epidemiological settings in sub-Saharan Africa.”*

5. From Lines 28 to 32, they seem to be contradictory. If the listed diseases are causes of morbidity and mortality in the target population, social and environmental issues can be causes of morbidity and mortality again. However, instead, they are determinants of health. This should be stated clearly.

We agree this can be clarified and now write (Lines 31-32):

*“…there is an urgent need to better understand the underlying social and environmental determinants of diseases that contribute significantly to morbidity and mortality”*

6. In Line 34, it started with "Access to adequate housing, a human right and a core aim of Sustainable Development Goal 11 [4], is a promising yet neglected topic in SSA". However, from Lines 35 through 38, the statement started with "Features of inadequate housing, including indoor air pollution, overcrowding, unsafe water and poor sanitation . . .". There is a disconnect here. It would have been better if the following statement continued to outline features of adequate housing instead of inadequate housing.

We thank the reviewer for their comment; however, since the two statements refer to different issues, we believe that the current wording is acceptable.

7. In Line 72, Materials and Methods, the section did not outline detailed information on the study's methods.

We thank the reviewer, but we clarify that detailed information on the study’s methods is provided from Line 72 to Line 194.

8. In Line 76, are these two the only seasons in the country?

We state in the manuscript (Line 77) that “*There are typically two rainy seasons each year*”.

9. In Line 82, there was information on the treatment of malaria as part of the study design

We thank the reviewer for this statement but are unclear what action is being requested. If the reviewer would like more information on the treatment of malaria, we confirm that this is already explained in Lines 123-124 “*If the thick blood smear was positive, the patient was diagnosed with malaria and managed according to national guidelines*” and Lines 82-84 “*For malaria, artemether-lumefantrine was adopted as the first-line treatment in 2006 in Tororo district, provided free of charge at public health facilities.”*

However, to increase clarity we now write (Lines 123-124):

*“If the thick blood smear was positive, the patient was diagnosed with malaria, treated with artemether-lumefantrine and managed according to national guidelines.”*

10. in Line 96, on “Enrollment and follow up of study participants”, no detailed information was provided on the study participants and the number of study participants in the previous study. How many were lost to follow-up?

We thank the reviewer and confirm that since data from the previous study are not analysed in the present study, information on the previous study is not included in the manuscript. All relevant study flow information for the present dataset, including loss to follow up, is provided.

11. In Line 186, “Enrollment and follow up of study participants”, out of the total participants, how many children and how many were adults? Or is it mother-child pair?

We confirm that this information is already present in Figure 1. However, we now write in Line 198:

*“A total of 531 participants (378 children and 153 adults)… [were enrolled]”.*

12. From Lines 254 to 320 in the discussion section, the presentations are results and not discussion. Discussion started from Line 322.

We thank the reviewer but note that it is standard practice to begin the Discussion with a summary of the main findings. Indeed, this is a requirement of STROBE.

13. In Line 374, how practicable can these recommendations be because the study area is rural. Most people are farmers who can barely afford basic daily needs much more, changing their housing patterns. Many other factors could result in gastrointestinal illness. What are the major factors responsible for this?

We thank the reviewer for their comment and confirm that current evidence suggests that housing is widely modernising in Tororo district (1) and more generally across urban and rural sub-Saharan Africa (2). Importantly, these changes are occurring with no input from the health sector and with little external funding or intervention; rather, they are community led and community funded as people invest in “incremental improvements” to their homes as incomes allow. These changes are occurring across the spectrum of the poorest communities as well as more affluent ones. In our study we simply document the relationship between these ongoing housing changes in the community with health outcomes such as malaria. Thus, the suggestion that community-led housing improvements may be beneficial for public health is entirely practicable.

Reviewer #2: This manuscript does an excellent job of demonstrating the link between modern housing and three major causes of child mortality in Sub-Saharan Africa: malaria, ARI, and gastrointestinal illness. Some of the my initial concerns regarding the sampling and of residence and habitants was well documented.
Authors have provided information on the data for the study as well as important findings and necessary documentation.
The article has been written in an intelligible fashion and the choice of words are appropriate for easy understanding.

We thank reviewer 2 and are delighted with their positive endorsement of our work.

Reviewer #3: The topic is one of global public health interest as housing conditions play a role in health and disease. However, the manuscript has some methodological flaws that can affect the interpretation of the findings. Some factors that can contribute to acute respiratory infection and gastrointestinal illness in house occupants such as biomass fuel and indoor cooking where either broadly classified or not identified. The method of house selection was not described, and some human laboratory procedures were poorly described and not referenced. The authors robustly used descriptive and inferential statistics and presented their findings in clear tables. The manuscript is presented in standard English though there are some sentence structure errors that need to be corrected. The authors can improve on the readability of the manuscript by ensuring there is a logical progression in their writing. For instance ethical approval should be obtained before community entry.
In all, the topic carries interest, but the authors have not demonstrated novelty in their research, and the conclusion does not tie up with the objective of the research.

We thank Reviewer 3 for their generally positive endorsement of our work. Individual comments are fully responded to below.

**Abstract**

The acronym CDC in line 8 should be written out in full for the first time

We have removed this acronym.

Recommendations (lines 19-21) is not drawn from the study.

We thank the reviewer for their comment. We are unclear what the reviewer would like to see, but we confirm that the study conclusions are carefully worded (e.g. the use of the word “may” in relation to the observed findings) and that we emphasise the need for more research in this area.

**Introduction**

Line 35 should be referenced: … is a promising yet neglected topic in SSA

We have added a reference.

**Materials and methods**

line 82-90 may fit better in the introduction

We thank the reviewer for their comment; however, we believe that the description of the study site belongs in the Methods.

The laboratory procedure for the thick blood smear examination should be referenced. Line 119

We have added a reference.

Line 110 and 120. It is unclear what the routine visits refers to. Is it the study participant visiting the facility or research assistants visiting the participants’ homes?

We have now clarified this by writing “*routine clinic visits*”.

Line 144: Write NASA out in full

We have made this change.

Line 115: Who were the field assistants? Were they trained and by who? How was any observer bias in laboratory analysis taken care of?

We have updated the manuscript to confirm that the field assistants were trained study staff.

We acknowledge the reviewer’s concern over observer bias in the laboratory analysis but would like to provide reassurance that this was not an issue. As is standard practice, laboratory staff had no means to identify participants. Additionally, this being a secondary data analysis, the study comparison could not have been known to these staff.

It is also not clear how the study households were selected. Was sample size calculated for this study? How were the number of modern or traditional houses required determined? Were no middle aged or older adults selected, as your selection criteria does not exclude them.

We thank the reviewer for their comments. We do already state that households were randomly selected but have further clarified this by writing (Lines 99-100) that households were “*randomly selected from an enumeration list in Nagongera sub-county”.*

Regarding sample size calculations, we now clarify (Lines 74-76) that the study was a secondary analysis of a cohort study “*designed to investigate optimal strategies for malaria control in Uganda using detailed longitudinal studies, with the sample size calculated according to this objective.”*

Since this was a secondary analysis, we did not recruit households based on whether they resided in modern or traditional homes.

We finally clarify that, although the average age of study participants was low, this is simply a reflection of the young population in Uganda (approximately 50% of the population is aged less than 15 years). We now clarify further that there were no age restrictions on enrolling participants (Lines 105-106):

*“All permanent residents of enrolled households, regardless of age, were screened and enrolled in the cohort study”.*

Line 159: Wealth categorization includes medium in line 159 but replaced with middle in table 3.

We have changed “medium” to “middle”.

There was no mention of consent sought from the head of household, considering the study setting was a rural African one.

We thank the reviewer and note that we do already include “willingness to participate” as the fourth inclusion criterion for households. However, we now confirm that consent was sought from the household head by writing (Lines 104-105): *“…the household head provided informed written consent to participate in the study”*

Level of statistical significance used in the study should be stated.

We thank the reviewer but have not stated level of statistical significance used; we report p-values and confidence intervals in full which gives all required information.

Line 191: It may be more appropriate to state the inter-quartile range when reporting median values.

We thank the reviewer but note that range is also acceptable to report.

**Results**

Line 126: Average time of going to bed only appeared for the first time here. It would be helpful to mention the study variables in the methodology.

We thank the reviewer and now clarify in the Methods (Lines 139-140) that:

“*The time that the room occupant went to bed the previous night was reported to study staff and recorded*.”

We confirm that all other exposure variables are mentioned in the Methods.

**Discussion**

Line 254: We identified and quantified a relationship between house construction and malaria…. House construction does not depict the outcome variable, and should be changed to something more appropriate

We thank the reviewer; however, we are not sure what their comment refers to. House construction is the exposure variable and malaria is one of our outcomes of interest.

Line 292: HBR was 30% lower in bedrooms with windows compared to windows.. Should be corrected.

We thank the reviewer and have corrected this typo.

Line 360 is an incomplete sentence.

We thank the reviewer but confirm that the line referenced is a complete sentence.

Once again, we thank the reviewers and editors for their efforts. We hope that these changes will meet the requirements for publication of our manuscript in *PLOS Global Public Health.*

Thank you again for considering our work.

Yours sincerely,

Mr Alex Musiime and Dr Lucy Tusting, on behalf of all authors

**References**

1.	Rek JC, Alegana V, Arinaitwe E, Cameron E, Kamya MR, Katureebe A, et al. Rapid improvements to rural Ugandan housing and their association with malaria from intense to reduced transmission: a cohort study. Lancet Planet Health. 2018;2:e83-e94.

2.	Tusting LS, Bisanzio D, Alabaster G, Cameron E, Cibulskis R, Davies M, et al. Mapping changes in housing in sub-Saharan Africa from 2000 to 2015. Nature. 2019;568:391-4.
